# Impact of the COVID-19 pandemic on severe non-SARS-CoV-2 community-acquired pneumonia in Reunion Island: a multicenter retrospective observational study, 2016–2021

**DOI:** 10.1038/s41598-023-40791-5

**Published:** 2023-08-28

**Authors:** Agathe Combe, David Kovacs, Axel de Mangou, Guillaume Miltgen, Nicolas Traversier, Olivier Belmonte, Olivier Simon, Charles Vidal, Nathalie Coolen-Allou, Jérôme Allyn, Nicolas Allou

**Affiliations:** 1 Intensive Care Unit, Centre Hospitalier Universitaire de La Réunion, Saint-Denis, Reunion Island France; 2 Microbiology, Centre Hospitalier Universitaire de La Réunion, Saint-Denis, Reunion Island France; 3 UMR Processus Infectieux en Milieu Insulaire Tropical, CNRS 9192, INSERM U1187, IRD 249, Centre Hospitalier Universitaire de La Réunion, Saint-Denis, Reunion Island France; 4 Intensive Care Unit, Centre Hospitalier Universitaire de La Réunion, Saint-Pierre, Reunion Island France; 5 Respiratory Medicine, Centre Hospitalier Universitaire de La Réunion, Saint-Denis, Reunion Island France; 6 Clinical Informatic Department, Centre Hospitalier Universitaire Felix Guyon, Saint-Denis, Reunion Island France; 7 Hôpital Felix Guyon, Réanimation Polyvalente, Bellepierre, 97405 Saint-Denis, France

**Keywords:** Clinical microbiology, Microbial communities

## Abstract

The Coronavirus 2019 (COVID-19) pandemic has had a considerable impact on the incidence of severe community-acquired pneumonia (CAP) worldwide. The aim of this study was to assess the early impact of the COVID-19 pandemic in the Reunion Island. This multicenter retrospective observational study was conducted from 2016 to 2021 in the hospitals of Reunion Island. The incidence of severe non-SARS-CoV-2 CAP, microorganisms, characteristics and outcomes of patients hospitalized in intensive care unit were compared between the pre-COVID-19 period (January 1, 2016 to February 29, 2020) and the early COVID-19 period (March 1, 2020 to October 31, 2021). Over the study period, 389 patients developed severe non-SARS-CoV-2 CAP. The incidence of severe non-SARS-CoV-2 CAP significantly decreased between the two periods (9.16 vs. 4.13 cases per 100,000 person-years). The influenza virus was isolated in 43.5% patients with severe non-SARS-CoV-2 CAP in the pre-COVID-19 period and in none of the 60 patients in the early COVID-19 period (P < 0.0001). The only virus that did not decrease was rhinovirus. *Streptococcus pneumoniae* was the most frequently isolated bacterial microorganism, with no significant difference between the two periods. In Reunion Island, the COVID-19 pandemic led to a significant decrease in the incidence of influenza, which likely explains the observed decrease in the incidence of severe non-SARS-CoV-2 CAP. The pandemic had no impact on the incidence of other viral and bacterial severe non-SARS-CoV-2 CAP. Monitoring influenza incidence is crucial now that COVID-19 control measures have been removed.

## Introduction

The coronavirus 2019 (COVID-19) pandemic caused by the severe acute respiratory syndrome coronavirus 2 (SARS-CoV-2)^1^ has had a subsantial influence on the incidence of lower respiratory tract infections worldwide. Following the emergence of the pandemic in December 2019, a significant decline in influenza cases was observed across Europe, the United States, Japan, Australia, and South America^2–5^. However, the impact of the COVID-19 pandemic on rhinovirus infections appears to have been less pronounced^6,7^. A recent study conducted in the United Kingdom (UK) suggests that the incidence of bacterial superinfections in patients with severe community-acquired pneumonia (CAP) has decreased during the pandemic^8^.

In the French overseas department of Reunion Island, in the Indian Ocean region, the first case of COVID-19 was diagnosed on March 11, 2020. As in metropolitan France, Reunion Island implemented a strict lockdown that lasted from March 17, 2020 to May 11, 2020. Control measures, including social distancing and mandatory mask-wearing in public, were officially initiated on the island on August, 2020. The vaccination campaign began on January 15, 2021. However, the vaccination rate in Reunion Island remained consistently lower than in metropolitan France throughout the pandemic (only two-thirds of Reunionese aged over 12 years were fully vaccinated^9^). Reunion Island was relatively spared during the first year of the COVID-19 pandemic, with only 9701 cases, 135 hospitalizations in intensive care unit (ICU), and 5.2 deaths per 100,000 inhabitants reported by January 24, 2021^10^. However, the epidemic intensified in the subsequent months. Prior to the pandemic, the main agent responsible for CAP in Reunion Island was influenza followed by *Streptococcus pneumoniae*, which was the opposite of the situation in metropolitan France^11^. Influenza circulated year-round, with a peak during the southern winter (when rainfall and temperatures are their lowest). The impact of the COVID-19 pandemic on severe non-SARS-CoV-2 CAP in Reunion Island might have been influenced by these epidemiological and climatic factors.

The aim of this study was to assess the early impact of the COVID-19 pandemic on severe non-SARS-CoV-2 CAP in Reunion Island.

## Methods

### Ethics

This observational study was approved by the French Ethics Committee of Infectious Disease and Tropical Medicine (CER-MIT, #COVID-2021-01) and was declared to the French National Commission for Data Protection and Liberties (CNIL, #2206739). Written and oral Informed Consent was obtained from all participants after they were given a written information notice about the process of data collection. All methods were performed in accordance with the declaration of Helsinki. The study complies with the Strengthening the Reporting of Observational studies in Epidemiology recommendations statement^12^.

### Selection of the study sample

We performed a retrospective chart review of all adult patients with suspected or confirmed low respiratory tract infection and hospitalized in ICU in one of the two university hospital centers of Reunion Island (Félix Guyon University Hospital and Saint Pierre University Hospital) between January 1, 2016 and October 31, 2021.

### Definitions

Community-acquired pneumonia was defined as pneumonia acquired outside hospital and diagnosed within 48 h of hospital admission. Diagnosis was established in the presence of a new lung infiltrate on chest x-ray or computed tomography scan together with one or more of the following symptoms and signs: fever > 38 °C, cough, expectoration, chest pain, dyspnea, and signs of invasion of the alveolar space^13^.

A severe case of CAP was defined as any patient with one of the major criteria and/or three or more of the minor criteria established by the American Thoracic Society^14^. Major criteria were septic shock requiring vasopressors and respiratory failure requiring mechanical ventilation. Minor criteria were respiratory rate > 30 breaths/min, PaO2/FIO2 ratio < 250, multilobar infiltrates, confusion or disorientation, blood urea nitrogen levels > 20 mg/dL, white blood cell count < 4 G/L, platelet count < 100 G/L, hypothermia < 36 °C, and hypotension requiring aggressive fluid resuscitation.

### Exclusion criteria

The exclusion criteria were: age < 18 years; lack of respiratory sample; nosocomial pneumonia; monomicrobial CAP caused by COVID-19; CAP of unknown etiology; and sanitary air evacuation from another island.

### Microbiological investigations

Blood and respiratory samples (sputum samples for non-intubated patients and tracheal or bronchoalveolar lavage for intubated patients) were collected from all patients. Microorganism identification was performed on both types of samples using Gram staining followed by culturing for definitive identification. Alternatively, identification was performed using matrix-assisted laser desorption ionization time-of-flight mass spectrometry.

Respiratory samples were tested by multiplex polymerase chain reaction (PCR) (Seegene Allplex™ respiratory panel, Eurobio-ingen, Les Ulis, France) for the following microorganisms: influenza type A (H1 and H3) and type B, respiratory syncytial virus, adenovirus, enterovirus, parainfluenza, human metapneumovirus, human bocavirus, rhinovirus, coronavirus (NL63, 229E, and OC43), *Chlamydia pneumoniae*, *Mycoplasma pneumoniae*, *Legionella* spp., *Haemophilus influenzae*, *S. pneumoniae*, *Bordetella pertussis*, and *B. parapertussis*.

Pneumococcal and Legionella urinary antigen tests were routinely performed on admission to ICU.

Serology for atypical respiratory microorganisms was performed at the physician’s discretion.

### Data collection

Data on patient characteristics (comorbidities, organ failure, organ support, and laboratory findings) were collected on hospital admission.

Data on outcomes of patients during ICU stay were also collected.

### Endpoints

The primary endpoint was the incidence of severe non-SARS-CoV-2 CAP in ICU patients in the pre-COVID-19 period (January 1, 2016 to February 29, 2020) and the early COVID-19 period (March 1, 2020 to October 31, 2021).

The secondary endpoints of the study included the following: the incidence of isolated microorganisms, the characteristics and outcomes (such as organ failure, organ support, duration of ICU stay, and mortality) of ICU patients with severe non-SARS-CoV-2 CAP in both the pre-COVID-19 period and the early COVID-19 period.

### Statistical analyses

Results were expressed as median [25th–75th percentile] for continuous variables and as percentage for categorical variables. Continuous variables were compared using the Mann–Whitney test or the unpaired Student’s t-test, as appropriate. Categorical variables were compared using the Chi-2 test or Fisher’s exact test, as appropriate. A value of P < 0.05 was considered significant. Statistical analyses were performed with SPSS for Windows version 15.0 (SPSS Inc, Chicago, Ill, USA).

## Results

### Study sample

**Figure 1:**
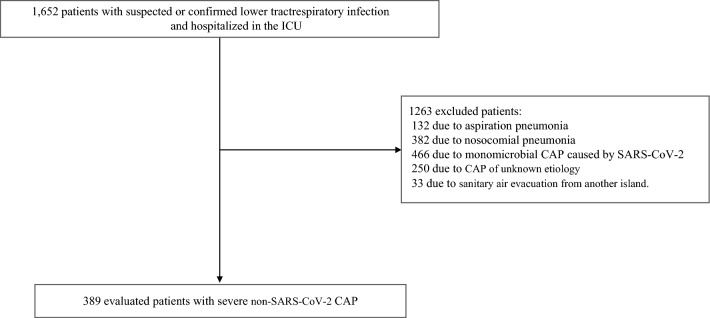
Selection of the study sample.

From January 1, 2016 to October 31, 2021, 1,652 patients were hospitalized in ICU for suspected or confirmed lower respiratory tract infection. Of these, 132 were excluded due to lack of respiratory sample; 382 due to nosocomial pneumonia; 466 due to monomicrobial CAP caused by COVID-19; 250 due to CAP of unknown etiology; and 33 due to a sanitary air evacuation from another island. The remaining 389 patients formed the cohort (Fig. 1).

### Incidence of severe non-SARS-CoV-2 community-acquired pneumonia

The COVID-19 pandemic led to a significant decrease in the incidence of severe non-SARS-CoV-2 CAP in Reunion Island. Indeed, severe non-SARS-CoV-2 CAP was diagnosed in 329 patients in the pre-COVID-19 period (9.16 cases per 100,000 person-years) compared to 60 patients in the early COVID-19 period (4.13 cases per 100,000 person-years).

### Incidence of microorganisms causing severe non-SARS-CoV-2 community-acquired pneumonia

**Figure 2:**
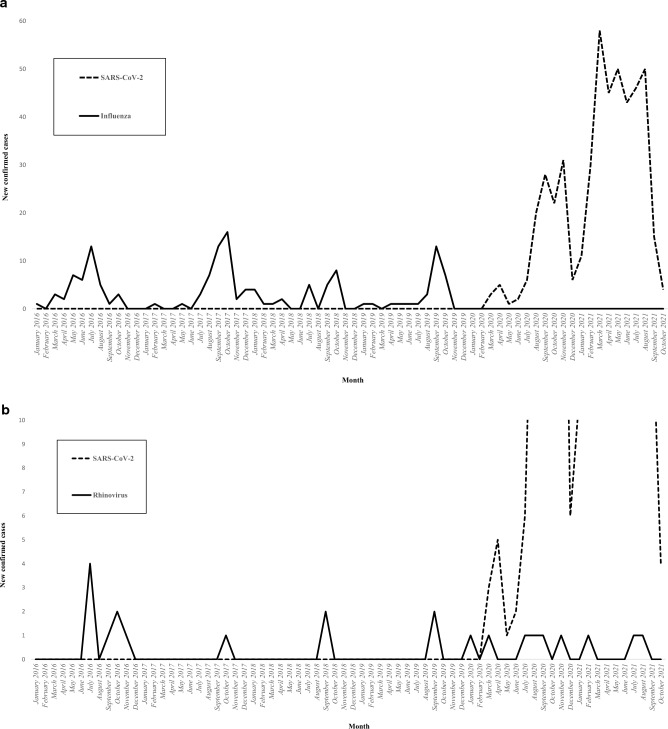

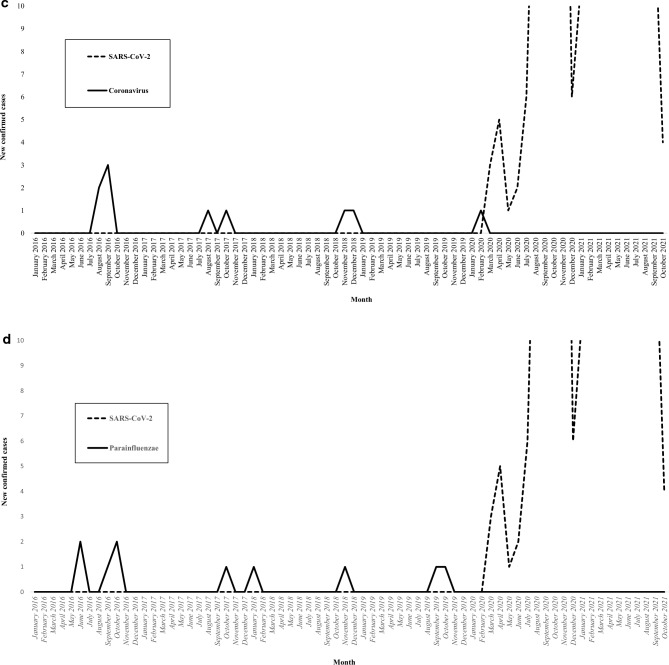

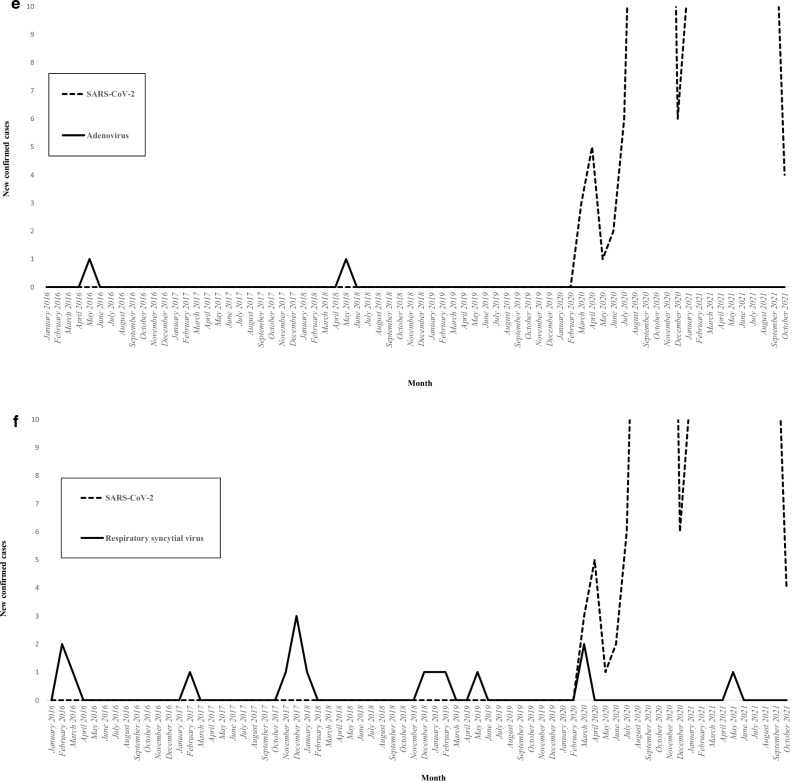

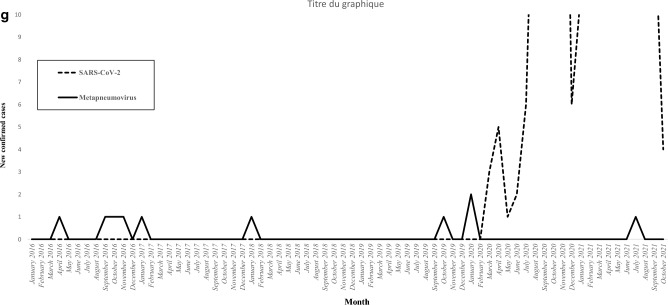
The incidence of viral microorganisms isolated in patients with severe non-SARS-CoV-2 community-acquired pneumonia according to the month of the year (2016–2021). (**a**) Influenza, (**b**) Rhinovirus, (**c**) Coronavirus, (**d**) Parainfluenzae, (**e**) Adenovirus, (**f**) Respiratory syncytial virus, (**g**) Metapneumovirus.

**Figure 3:**
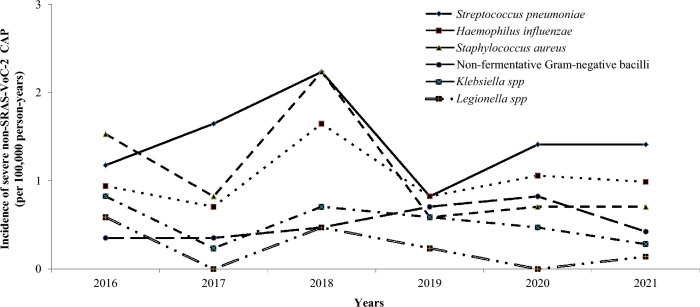
The incidence of the different bacterial microorganisms isolated in patients with severe non-SARS-CoV-2 community-acquired pneumonia according to the years (2016–2021).

Figure 2 illustrates the incidence of viral microorganisms isolated in patients with severe non-SARS-CoV-2 CAP according to the month of the year. The influenza virus was isolated during 34 out of 50 months (68%) in the pre-COVID-19 period, with one annual epidemic peak (Fig. 2a). While the influenza virus was isolated in 143 out of 329 patients with severe non-SARS-CoV-2 CAP (43.5%) in the pre-COVID-19 period, it was not isolated in any of the 60 patients with severe non-SARS-CoV-2 CAP in the early COVID-19 period (P = 0.0001). The only viral CAPs that did not decrease in incidence with the COVID-19 epidemic were those caused by rhinovirus (Fig. 2b). In fact, the incidence of rhinovirus tended to increase during the pandemic since it was isolated in 15 out of 329 patients with severe non-SARS-CoV-2 CAP in the pre-COVID-19 period (4.6%) compared to 7 out of 60 patients with severe non-SARS-CoV-2 CAP in the early COVID-19 period (11.7%, P = 0.06). No significant difference was observed in the incidence of other respiratory viruses between the two periods (Fig. 2c–g). The incidence of the different bacterial microorganisms isolated in patients with severe non-SARS-CoV-2 CAP varied across years. However, this incidence was not affected by the COVID-19 epidemic (Fig. 3). The incidence of severe non-SARS-CoV-2 CAP due to *S. pneumoniae* was 1.47 cases per 100,000 person-years in the pre-COVID-19 period compared to 1.41 cases per 100,000 person-years in the early COVID-19 period. Figure 3 presents the incidence of the main bacterial microorganisms isolated in patients with severe non-SARS-CoV-2 CAP according to the year.

### Characteristics and outcomes of patients hospitalized in intensive care unit for severe non-SARS-CoV-2 community-acquired pneumonia

The median age of patients was 61 [51–71] years (61 [51–71] years in the pre-COVID-19 period and 61 [53–70] years in the early COVID-19 period, P = 0.954). The median Simplified Acute Physiology Score II score on admission was 45 [33–59] (44 [33–58] in the pre-COVID-19 period and 48 [31–61] in the early COVID-19 period, P = 0.322). There was no significant difference in the median delay from symptom onset to ICU admission between the pre-COVID-19 period (3 [1–6.5]) and the early COVID-19 period (3 [1–6.5], P = 0.96).

The use of high-flow nasal oxygen therapy was significantly higher in the early COVID-19 period than in the pre-COVID-19 period (36.7 vs. 15.5, P < 0.001). A significant increase was also observed in the use of invasive mechanical ventilation (86,7 vs. 67.2%, P = 0.002), renal replacement therapy (35% vs. 20.7%, P = 0.019), and catecholamines (76.7% vs. 57.1%, P = 0.004).

Total bilirubin levels increased significantly with the pandemic (11 [7–19] µmol/L in the pre-COVID-19 period and 15 [8.5–22.5] µmol/L in the early COVID-19 period, P = 0.037). No significant differences were observed in other laboratory measures between the two periods.

The median duration of mechanical ventilation was not significantly different between the pre-COVID-19 period (5 [4–11]) and the early COVID-19 period (6 [2–13], P = 0.54). Likewise, the median duration of ICU stay did not significantly vary with the pandemic (7 [4–14]) for the pre-COVID-19 period and 9 [5–15] for the early COVID-19 period, P = 0.58). In-ICU mortality was 28.6% in the pre-COVID-19 period and 28.3% in the early COVID-19 period (P = 0.99).

Table 1 presents the characteristics of patients on admission to ICU and the outcomes of patients during ICU stay.

**Table 1:** Baseline patient characteristics and organ failure failure during in ICU stay.

	Total	Before COVID-19	During COVID-19	*P*-value
	(n = 389)	(n = 329)	(n = 60)	
Comorbidities				
Age (years)	61 [51–71]	61 [50–71]	61 [53–70]	0.954
Male sex	235 (60.4)	197 (59.9)	38 (63.3)	0.668
Body mass index (kg/m^2^)	24 [20.6–28.7]	24 [21.1–28.9]	23.1 [17–28]	0.061
Immunodepression	47 (12.1)	36 (10.9)	11 (18.3)	0.129
Chronic obstructive pulmonary disease	135 (34.7)	107 (32.5)	18 (30)	0.639
Asthma	40 (10.3)	29 (8.8)	5 (8.3)	0.736
Arterial hypertension	181 (46.5)	153 (46.5)	28 (46.7)	1
Chronic renal failure with dialysis	24 (6.2)	20 (6.1)	4 (6.7)	0.775
Diabetes mellitus	132 (33.9)	110 (33.4)	22 (36.7)	0.658
Liver cirrhosis	16 (4.1)	16 (4.9)	0	1
Cancer < 4 month	27 (6.9)	22 (6.7)	5 (8.3)	0.587
History of congestive heart failure	76 (19.5)	68 (20.7)	8 (13.3)	0.218
Alcohol use disorder	91 (23.4)	80 (24.3)	11 (18.3)	0.407
Pregnancy	7 (1.8)	6 (1.8)	1 (1.7)	1
Corticosteroids	46 (11.8)	42 (12.8)	4 (6.7)	0.275
Duration of symptoms before ICU admission (days)	3 [1–6.5]	3 [1–6.5]	3 [1–6.5]	0.958
Organ failure/organ supports at ICU admission				
SAPS 2	45 [33–59]	44 [33–58]	48 [31–61]	0.322
Glasgow Coma Scale score	15 [11–15]	15 [10–15]	15 [12–15]	0.969
PaO2/FiO2 ratio (mmHg)	150 [96–202]	152 [100–217]	108 [72–176]	0.003
Temperature (°C)	37.9 [36.7–38.9]	38 [36.7–39]	37.8 [36.7–38.5]	0.422
ARDS	286 (73.5)	247 (75.1)	39 (65)	0.113
Sepsis	91 (23.4)	80 (24.3)	11 (18.3)	0.407
Laboratory findings at ICU admission				
Creatinine level (µmol/L)	109 [71–187]	108 [70–177]	126 [74–223]	0.149
Total bilirubin level (µmol/L)	12 [7–21]	11 [7–19]	15 [8.5–22.5]	0.037
Prothrombin time (%)	70 [52–83]	70 [53–84]	64 [48–81]	0.096
Platelet count (G/L)	178 [122–247]	175 [123–246]	184 [93–290]	0.941
Leucocytes count (G/L)	9.7 [4.6–14.8]	10.1 [5.1–14.9]	7.9 [4.5–14.5]	0.13
Lactatemia level (mmol/L)	1.7 [1–3.3]	1.8 [1–3.2]	1.5 [1–4]	0.25
Creatine phosphokinase (mg/dL)	238 [101–685]	246 [101–685]	187 [83–622]	0.602
Hemoglobin level (g/dL)	11.5 [8.1–13.2]	11.8 [9–13.3]	10.2 [9.3–12]	0.12
Alanine aminotransferase level (UI/L)	30 [17–60]	30 [18–62]	27 [14–58]	0.331
Troponin level (ng/dL)	38 [15–120]	38 [16–112]	42 [12–146]	0.827
C-reactive Protein level (mg/L)	152 [56–311]	170 [70–324]	109 [23–279]	0.065
Organ failure/organ supports during in ICU stay				
Extracorporeal membrane oxygenation	25 (6.4)	20 (6.1)	5 (8.3)	0.564
Invasive mechanical ventilation	273 (70.2)	221 (67.2)	52 (86.7)	0.002
High-flow nasal oxygenation	73 (18.8)	51 (15.5)	22 (36.7)	< 0.001
Renal replacement therapy	89 (22.9)	68 (20.7)	21 (35)	0.019
Catecholamines	234 (60.2)	188 (57.1)	46 (76.7)	0.004

*ARDS* acute respiratory distress syndrome, *ICU* intensive care unit, *SAPS 2* simplified acute physiology score.

## Discussion

Our study is the first assessment of the impact of the COVID-19 pandemic on severe non-SARS-CoV-2 CAP in Reunion Island. We observed a significant decrease in the incidence of severe non-SARS-CoV-2 CAP between the pre-COVID-19 period and the early COVID-19 period. The incidence of severe non-SARS-CoV-2 CAP caused by influenza also declined between this time. The only virus that did not decrease in incidence with the COVID-19 pandemic was the rhinovirus. *Streptococcus pneumoniae* was the most frequently isolated bacterial microorganism in both the pre- and early COVID-19 periods.

The observed decrease in the incidence of severe non-SARS-CoV-2 CAP in Reunion Island aligns with findings from Asia, Europe, and South America, where hospitalizations or consultations for CAP decreased during the COVID-19 pandemic^15–17^. This reduction is generally attributed to the implementation of control measures worldwide such as social distancing and mask mandates, aimed at combating COVID-19^18–20^. Another possible explanation is the lower use of healthcare facilities due to fears of contracting COVID-19. The study by Wu et al., which reported an excess of non-COVID-19-related deaths at home during the pandemic, provides support for this hypothesis^21^.

Before the COVID-19 pandemic, the main microorganism responsible for severe non-SARS-CoV-2 CAP in Reunion Island was influenza followed by *S. pneumoniae*-a finding consistent with studies utilizing PCR for microorganism identification, as was done in our study^22–24^. However, the epidemiological dynamics of severe non-SARS-CoV-2 CAP in Reunion Island were clearly impacted by the COVID-19 pandemic. There was a significant decrease in the incidence of severe non-SARS-CoV-2 CAP due to influenza, which consequently explains the overall decrease of the incidence of severe non-SARS-CoV-2 CAP. Thus, influenza was present 68% of the time between January 2016 and December 2019 (with one yearly epidemic peak during the austral winter from May to October), while no cases of severe non-SARS-CoV-2 CAP due to influenza were diagnosed after the onset of the pandemic. Registry-based population studies conducted in other countries reported similar findings^2,18^. The slight increase in vaccine coverage from the pre-COVID period (33% in 2016 and 2019) to the early COVID-19 period (41% in 2020)^25,26^ appears insufficient to explain these results, particularly considering that it remains well below the optimal vaccination coverage threshold of 75% recommended by the World Health Organization (WHO)^27^.

The drastic decline in influenza worldwide is likely the result of the control measures against COVID-19 rather than the pandemic itself. Indeed, many cases of coinfections with COVID-19 and influenza were reported at the beginning of the pandemic^28^. Moreover, the removal of social distancing measures in Mayotte and Reunion Island in September 2021 (during the southern winter) resulted in the resurgence of severe CAP due to influenza^29^. Consequently, close monitoring of influenza incidence is crucial on Reunion Island now that COVID-19 control measures have been fully removed. Notably, the COVID-19 pandemic did not impact the incidence of severe non-SARS-CoV-2 CAP due to rhinovirus in Reunion Island. The persistence of rhinovirus could be explained by the fact that the control measures implemented to fight COVID-19 had an impact on enveloped viruses but not on non-enveloped ones^30^. Another possible explanation is that surgical masks are less effective against rhinovirus^31^. It is also likely that the rhinovirus continued to circulate among school children as these were less impacted by the lockdown and social distancing measures^7,30^. The persistence of rhinovirus, however, is a source of concern regardless of its cause. Indeed, this virus is now viewed as a truly pathogenic microorganism^32–34^, and its economic and social impact has been shown to be as significant as that of influenza^35^.

The incidence of severe non-SARS-CoV-2 CAP due to *S. pneumoniae* or *S. aureus* was not significantly impacted by the COVID-19 pandemic in Reunion Island. This is surprising considering the observed decrease in the incidence of severe non-SARS-CoV-2 CAP due to influenza. Indeed, severe respiratory bacterial superinfections are generally associated with influenza, as demonstrated for *S. aureus*^36^. The fact that the incidence of severe non-SARS-CoV-2 CAP due to *S. pneumoniae* or *S. aureus* remained relatively stable despite the decrease in the incidence of influenza suggests that these bacterial CAPs are not always superinfections of influenza. However, it is worth noting that studies conducted in Hong Kong^37^ and England^8^ did observe a decrease in severe CAP due to *S. pneumoniae* following the implementation of COVID-19 control measures.

Modeling studies had anticipated a reduction in the transmissibility of COVID-19 in tropical areas with increased temperatures^38^. Yet, in Mayotte, a French overseas territory located close to Reunion Island, the largest wave of COVID-19 occurred in February 2021, a months with the highest temperatures and rainfall^39,40^. Thus, the number of cases reported in only one month in February 2021 was 8,630 compared to 8,231 cases prior to February 1, 2021.^38^. Likewise, no impact of the summer temperature increase on the trajectory of the COVID-19 pandemic was observed in Iran^41^. In Brazil, factors related to human development (household income, education, and level of health) were found to have a greater impact on the spread of COVID-19 than climatic factors^42^.

The use of high-flow nasal oxygen therapy significantly increased during the COVID-19 pandemic in Reunion Island. This can be explained by intensivists increasingly adopting to this technique for ñamaging patients with severe CAP^43^ after noting its association with improved prognosis in patients with severe COVID-19 pneumonia^44^.

Our study has some limitations. First, there may be biases introduced due to its retrospective nature. Second, our study only focused on patients hospitalized in ICU, so we cannot draw conclusion regarding the impact of the COVID-19 pandemic on the incidence of mild CAP. This is unfortunate as some studies suggest that the impact of the pandemic on mild CAP was even greater than on severe CAP^45^. Lastly, PCR analysis was predominantly performed on nasopharyngeal swabs samples, as many evaluated patients were not on invasive mechanical ventilation. We can suppose that our results would have been different if a higher proportion of deep respiratory samples (endotracheal aspiration, bronchoalveolar lavage, etc.) had been used. However, recent studies comparing the diagnostic performance of PCR on nasopharyngeal and deep respiratory samples have found no significant differences between the two techniques, except for certain microorganisms like Legionella pneumophilia^24^.

A major strength of our study is that we evaluated the incidence of all microorganisms responsible for severe non-SARS-CoV-2 CAP over a 20-month period during the COVID-19 pandemic. In contrast, epidemiological studies assessing the impact of the pandemic on severe non-SARS-CoV-2 CAP were conducted over a short period, used national registries, and did not investigate respiratory pathogens other than influenza (e.g., rhinovirus, *S. aureus*, *S. pneumoniae*, etc.)^2,4^. Another strength of our study is that the sample was exhaustive, as all patients with confirmed severe non-SARS-CoV-2 CAP in Reunion Island were managed in the two university hospitals included in the analysis. Furthermore, in Reunion Island, there were no restrictions on ICU admission. Accordingly, our data can be considered representative of the dynamics of severe non-SARS-CoV-2 CAP in Reunion Island during the early stages of the COVID-19 pandemic.

Future studies should be conducted in Reunion Island to evaluate the impact of the COVID-19 pandemic on severe non-SARS-CoV-2 CAP beyond the period included in this study.

## Conclusions

In Reunion Island, the COVID-19 pandemic led to a significant decrease in the incidence of influenza, which likely explains the observed decrease in the incidence of severe non-SARS-CoV-2 CAP. By contrast, the pandemic had no impact on the incidence of other viral and bacterial severe non-SARS-CoV-2 CAP. Monitoring influenza incidence is crucial now that COVID-19 control measures have been removed.

## Data Availability

The dataset used in the current study are available from the corresponding author.

## Abbreviations

COVID-19: Coronavirus 2019
CAP: Community-acquired pneumonia
ICU: Intensive care unit
PCR: Polymerase chain reaction
